# Ethyl 2-[5-(4-chloro­phen­yl)-1-(4-fluoro­phen­yl)-1*H*-pyrazol-3-yl]-4-methyl­thia­zole-5-carboxyl­ate

**DOI:** 10.1107/S1600536810042066

**Published:** 2010-10-23

**Authors:** Wan-Sin Loh, Hoong-Kun Fun, R. Venkat Ragavan, V. Vijayakumar, S. Sarveswari

**Affiliations:** a X-ray Crystallography Unit, School of Physics, Universiti Sains Malaysia, 11800 USM, Penang, Malaysia; b Organic Chemistry Division, School of Advanced Sciences, VIT University, Vellore 632 014, India

## Abstract

In the title compound, C_22_H_17_ClFN_3_O_2_S, the pyrazole ring is approximately planar with a maximum deviation of 0.001 (4) Å and makes dihedral angles of 4.95 (19), 35.78 (18) and 54.73 (18)° with the thia­zole, fluoro­benzene and chloro­benzene rings, respectively. In the crystal, inter­molecular C—H⋯O hydrogen bonds link the mol­ecules into chains along the *a* axis.

## Related literature

For background to pyrazole derivatives and their anti­microbial activity, see: Ragavan *et al.* (2009 ▶, 2010 ▶). For bond-length data, see: Allen *et al.* (1987 ▶). For a related structure, see: Loh *et al.* (2010 ▶). For the stability of the temperature controller used in the data collection, see: Cosier & Glazer (1986 ▶).
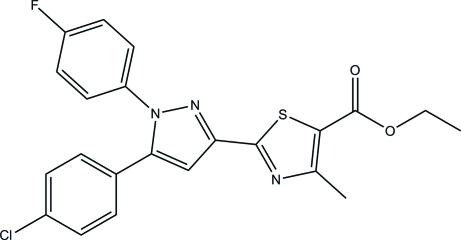


## Experimental

### 

#### Crystal data

C_22_H_17_ClFN_3_O_2_S
*M* *_r_* = 441.90
Monoclinic, 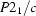

*a* = 12.0296 (5) Å
*b* = 19.4428 (6) Å
*c* = 9.5847 (3) Å
β = 112.922 (1)°
*V* = 2064.74 (12) Å^3^
*Z* = 4
Mo *K*α radiation
μ = 0.32 mm^−1^
*T* = 100 K
0.42 × 0.17 × 0.08 mm

#### Data collection

Bruker SMART APEXII CCD area-detector diffractometer
Absorption correction: multi-scan (*SADABS*, Bruker, 2009 ▶) *T*_min_ = 0.878, *T*_max_ = 0.976
30630 measured reflections
4697 independent reflections
3944 reflections with *I* > 2σ(*I*)
*R*_int_ = 0.043

#### Refinement

*R*[*F* ^2^ > 2σ(*F* ^2^)] = 0.056
*wR*(*F* ^2^) = 0.163
*S* = 1.24
4697 reflections
273 parameters
H-atom parameters constrained
Δρ_max_ = 0.63 e Å^−3^
Δρ_min_ = −0.53 e Å^−3^

### 

Data collection: *APEX2* (Bruker, 2009 ▶); cell refinement: *SAINT* (Bruker, 2009 ▶); data reduction: *SAINT*; program(s) used to solve structure: *SHELXTL* (Sheldrick, 2008 ▶); program(s) used to refine structure: *SHELXTL*; molecular graphics: *SHELXTL*; software used to prepare material for publication: *SHELXTL* and *PLATON* (Spek, 2009 ▶).

## Supplementary Material

Crystal structure: contains datablocks global, I. DOI: 10.1107/S1600536810042066/fj2354sup1.cif

Structure factors: contains datablocks I. DOI: 10.1107/S1600536810042066/fj2354Isup2.hkl

Additional supplementary materials:  crystallographic information; 3D view; checkCIF report

## Figures and Tables

**Table 1:** Hydrogen-bond geometry (Å, °)

*D*—H⋯*A*	*D*—H	H⋯*A*	*D*⋯*A*	*D*—H⋯*A*
C15—H15*A*⋯O2^i^	0.93	2.48	3.251 (5)	141

Symmetry code: (i) 
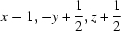
.

**Fig. 1.**
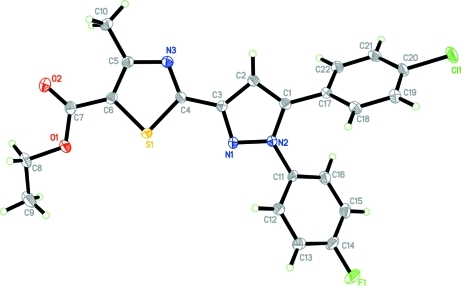
The molecular structure of the title compound, showing 50% probability displacement ellipsoids and the atom-numbering scheme.

**Fig. 2.**
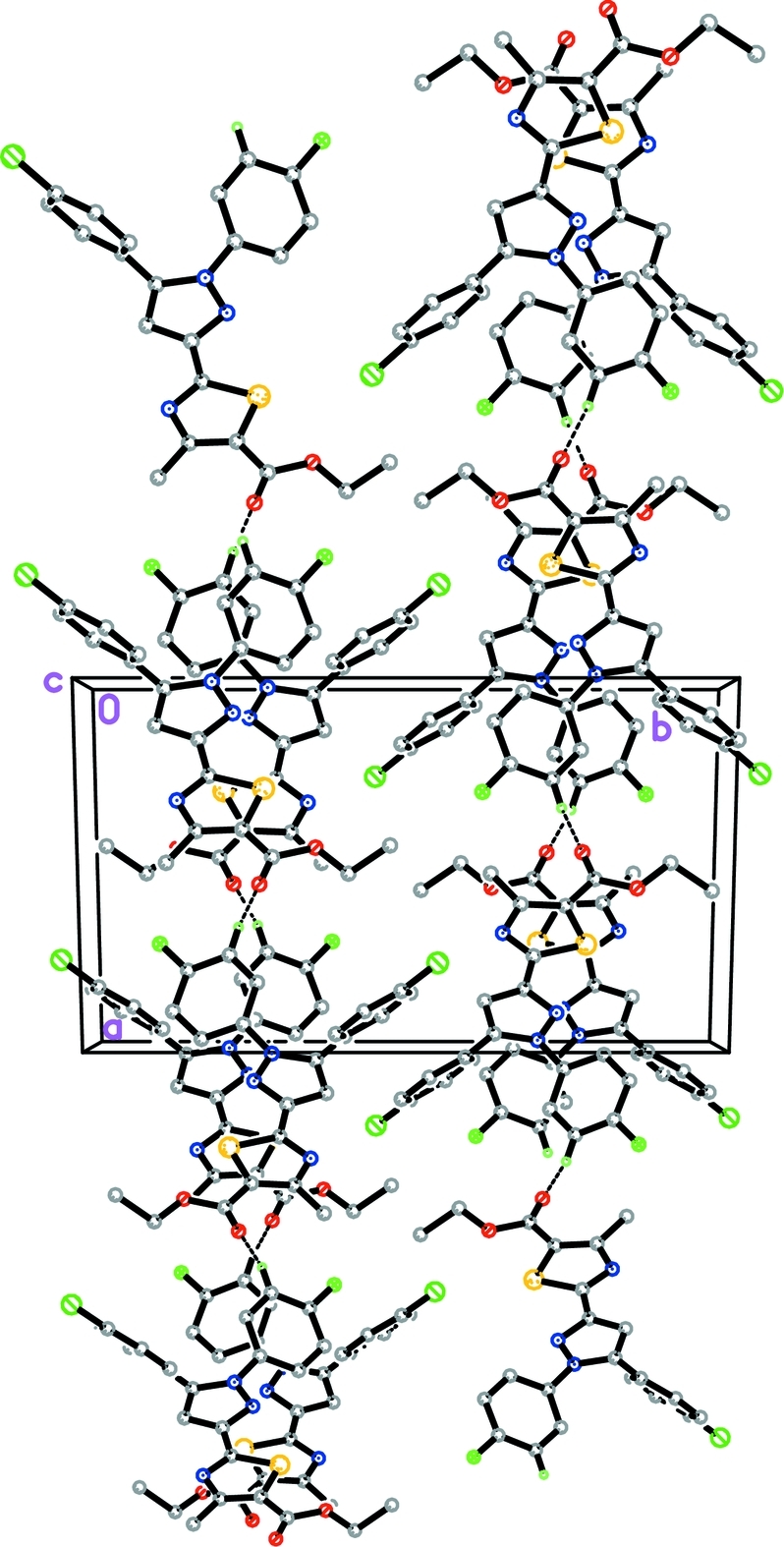
The crystal packing of the title compound, showing one-dimensional chains along the a axis. H atoms not involved in the intermolecular interactions (dashed lines) have been omitted for clarity.

## supplementary crystallographic information

### Comment

Antibacterial and antifungal activities of azoles are most widely studied and some of them are in clinical practice as anti-microbial agents. However, the azole-resistant strains had led to the development of new antimicrobial compounds. In particular pyrazole derivatives are extensively studied and used as antimicrobial agents. Pyrazole is an important class of heterocyclic compounds and many pyrazole derivatives are reported to have a broad spectrum of biological properties, such as anti-inflammatory, antifungal, herbicidal, anti-tumour, cytotoxic, molecular modelling and antiviral activities. Pyrazole derivatives also act as anti-angiogenic agents, A3 adenosine receptor antagonists, neuropeptide YY5 receptor antagonists, kinase inhibitor for treatment of type 2 diabetes, hyperlipidemia, obesity and thrombopiotinmimetics. Recently urea derivatives of pyrazoles have been reported as potent inhibitors of p38 kinase. Since the high electronegativity of halogens (particularly chlorine and fluorine) in the aromatic part of the drug molecules play an important role in enhancing their biological activity, we are interested to have 4-fluoro or 4-chloro substitution in the aryls of 1,5-diaryl pyrazoles. As part of our on-going research aiming the synthesis of new antimicrobial compounds, we have reported the synthesis of novel pyrazole derivatives and their microbial activities (Ragavan *et al.*, 2009;2010).

The title compound consists of four rings, namely pyrazole (C1–C3/N1/N2), thiazole (C4/N3/C5/C6/S1), fluorophenyl (C11–C16/F1) and chlorophenyl (C17–C22/Cl1) rings (Fig. 1). The pyrazole ring is approximately planar with a maximum deviation of 0.001 (4) Å at atom C1 and makes dihedral angles of 4.95 (19), 35.78 (18) and 54.73 (18)° with the thiazole, fluorophenyl and chlorophenyl rings, respectively. Bond lengths (Allen *et al.*, 1987) and angles are within the normal ranges and are comparable to the related structure (Loh *et al.*, 2010).

In the crystal packing (Fig. 2), intermolecular C15—H15A···O2 hydrogen bonds link the molecules into one-dimensional chains along the *a* axis.

### Experimental

The compound has been synthesized using the method available in the literature (Ragavan *et al.*, 2010) and recrystallized using the ethanol-chloroform 1:1 mixture. Yield: 81%. *M.p.*: 411.3–413 K.

### Refinement

All H atoms were positioned geometrically with the bond length of C–H being 0.93 to 0.97 Å and were refined using a riding model, with *U*_iso_(H) = 1.2 or 1.5 *U*_eq_(C). A rotating group model was applied to the methyl groups.

### Crystal data

**Table:**

C_22_H_17_ClFN_3_O_2_S	*F*(000) = 912
*M**_r_* = 441.90	*D*_x_ = 1.422 Mg m^−^^3^
Monoclinic, *P*2_1_/*c*	Mo *K*α radiation, λ = 0.71073 Å
Hall symbol: -P 2ybc	Cell parameters from 9879 reflections
*a* = 12.0296 (5) Å	θ = 2.8–32.9°
*b* = 19.4428 (6) Å	µ = 0.32 mm^−^^1^
*c* = 9.5847 (3) Å	*T* = 100 K
β = 112.922 (1)°	Plate, colourless
*V* = 2064.74 (12) Å^3^	0.42 × 0.17 × 0.08 mm
*Z* = 4	

### Data collection

**Table:**

Bruker SMART APEXII CCD area-detector diffractometer	4697 independent reflections
Radiation source: fine-focus sealed tube	3944 reflections with *I* > 2σ(*I*)
graphite	*R*_int_ = 0.043
φ and ω scans	θ_max_ = 27.5°, θ_min_ = 1.8°
Absorption correction: multi-scan (*SADABS*, Bruker, 2009)	*h* = −15→15
*T*_min_ = 0.878, *T*_max_ = 0.976	*k* = −25→25
30630 measured reflections	*l* = −12→12

### Refinement

**Table:**

Refinement on *F*^2^	Primary atom site location: structure-invariant direct methods
Least-squares matrix: full	Secondary atom site location: difference Fourier map
*R*[*F*^2^ > 2σ(*F*^2^)] = 0.056	Hydrogen site location: inferred from neighbouring sites
*wR*(*F*^2^) = 0.163	H-atom parameters constrained
*S* = 1.24	*w* = 1/[σ^2^(*F*_o_^2^) + (0.*P*)^2^ + 9.3055*P*] where *P* = (*F*_o_^2^ + 2*F*_c_^2^)/3
4697 reflections	(Δ/σ)_max_ < 0.001
273 parameters	Δρ_max_ = 0.63 e Å^−^^3^
0 restraints	Δρ_min_ = −0.53 e Å^−^^3^

### Special details

**Table:**

Experimental. The crystal was placed in the cold stream of an Oxford Cryosystems Cobra open-flow nitrogen cryostat (Cosier & Glazer, 1986) operating at 100.0 (1) K.
Geometry. All e.s.d.'s (except the e.s.d. in the dihedral angle between two l.s. planes) are estimated using the full covariance matrix. The cell e.s.d.'s are taken into account individually in the estimation of e.s.d.'s in distances, angles and torsion angles; correlations between e.s.d.'s in cell parameters are only used when they are defined by crystal symmetry. An approximate (isotropic) treatment of cell e.s.d.'s is used for estimating e.s.d.'s involving l.s. planes.
Refinement. Refinement of *F*^2^ against ALL reflections. The weighted *R*-factor *wR* and goodness of fit *S* are based on *F*^2^, conventional *R*-factors *R* are based on *F*, with *F* set to zero for negative *F*^2^. The threshold expression of *F*^2^ > σ(*F*^2^) is used only for calculating *R*-factors(gt) *etc*. and is not relevant to the choice of reflections for refinement. *R*-factors based on *F*^2^ are statistically about twice as large as those based on *F*, and *R*- factors based on ALL data will be even larger.

### Fractional atomic coordinates and isotropic or equivalent isotropic displacement parameters (Å^2^)

**Table:**

	*x*	*y*	*z*	*U*_iso_*/*U*_eq_	
S1	0.28859 (8)	0.28472 (4)	−0.01128 (10)	0.0162 (2)	
Cl1	−0.24355 (9)	−0.04920 (5)	0.40468 (12)	0.0288 (2)	
F1	−0.2939 (2)	0.38188 (13)	0.2896 (3)	0.0373 (6)	
O1	0.4449 (2)	0.36177 (13)	−0.1138 (3)	0.0239 (6)	
O2	0.5434 (2)	0.27417 (14)	−0.1712 (3)	0.0247 (6)	
N1	0.0936 (3)	0.24138 (15)	0.0961 (3)	0.0161 (6)	
N2	0.0143 (3)	0.21207 (15)	0.1472 (3)	0.0154 (6)	
N3	0.3202 (3)	0.15335 (15)	0.0001 (4)	0.0176 (6)	
C1	0.0322 (3)	0.14214 (17)	0.1675 (4)	0.0160 (7)	
C2	0.1269 (3)	0.12630 (18)	0.1269 (4)	0.0165 (7)	
H2A	0.1608	0.0832	0.1279	0.020*	
C3	0.1616 (3)	0.18936 (18)	0.0837 (4)	0.0164 (7)	
C4	0.2558 (3)	0.20259 (17)	0.0272 (4)	0.0155 (7)	
C5	0.4011 (3)	0.18060 (18)	−0.0546 (4)	0.0178 (7)	
C6	0.3963 (3)	0.25092 (18)	−0.0694 (4)	0.0165 (7)	
C7	0.4701 (3)	0.29491 (19)	−0.1238 (4)	0.0185 (7)	
C8	0.5145 (4)	0.4118 (2)	−0.1592 (5)	0.0281 (9)	
H8A	0.5329	0.3938	−0.2422	0.034*	
H8B	0.5898	0.4220	−0.0750	0.034*	
C9	0.4385 (4)	0.4758 (2)	−0.2080 (5)	0.0326 (10)	
H9A	0.4861	0.5124	−0.2229	0.049*	
H9B	0.4099	0.4887	−0.1310	0.049*	
H9C	0.3708	0.4669	−0.3010	0.049*	
C10	0.4855 (4)	0.1330 (2)	−0.0880 (5)	0.0257 (9)	
H10A	0.4783	0.1399	−0.1903	0.039*	
H10B	0.4654	0.0863	−0.0754	0.039*	
H10C	0.5669	0.1424	−0.0196	0.039*	
C11	−0.0686 (3)	0.25574 (18)	0.1786 (4)	0.0150 (7)	
C12	−0.0298 (3)	0.32104 (18)	0.2356 (4)	0.0179 (7)	
H12A	0.0474	0.3358	0.2498	0.021*	
C13	−0.1070 (3)	0.36415 (19)	0.2714 (4)	0.0207 (8)	
H13A	−0.0829	0.4083	0.3082	0.025*	
C14	−0.2197 (4)	0.3401 (2)	0.2510 (5)	0.0247 (8)	
C15	−0.2614 (3)	0.2759 (2)	0.1924 (5)	0.0242 (8)	
H15A	−0.3383	0.2613	0.1796	0.029*	
C16	−0.1848 (3)	0.2336 (2)	0.1528 (4)	0.0215 (8)	
H16A	−0.2113	0.1908	0.1093	0.026*	
C17	−0.0370 (3)	0.09629 (17)	0.2271 (4)	0.0162 (7)	
C18	−0.0471 (3)	0.10914 (19)	0.3647 (4)	0.0207 (8)	
H18A	−0.0105	0.1478	0.4208	0.025*	
C19	−0.1115 (3)	0.06460 (19)	0.4186 (5)	0.0229 (8)	
H19A	−0.1185	0.0732	0.5103	0.027*	
C20	−0.1653 (3)	0.00709 (18)	0.3334 (5)	0.0205 (8)	
C21	−0.1571 (3)	−0.00729 (18)	0.1970 (5)	0.0217 (8)	
H21A	−0.1939	−0.0461	0.1416	0.026*	
C22	−0.0923 (3)	0.03780 (18)	0.1440 (4)	0.0204 (8)	
H22A	−0.0858	0.0289	0.0521	0.025*	

### Atomic displacement parameters (Å^2^)

**Table:**

	*U*^11^	*U*^22^	*U*^33^	*U*^12^	*U*^13^	*U*^23^
S1	0.0197 (4)	0.0132 (4)	0.0191 (5)	0.0005 (3)	0.0115 (3)	−0.0001 (3)
Cl1	0.0279 (5)	0.0246 (5)	0.0372 (6)	−0.0057 (4)	0.0161 (4)	0.0090 (4)
F1	0.0383 (14)	0.0349 (14)	0.0523 (18)	0.0144 (11)	0.0324 (13)	0.0036 (12)
O1	0.0305 (14)	0.0161 (12)	0.0328 (16)	−0.0035 (11)	0.0209 (13)	0.0009 (11)
O2	0.0246 (14)	0.0259 (14)	0.0306 (16)	−0.0008 (11)	0.0184 (12)	0.0002 (12)
N1	0.0176 (14)	0.0160 (14)	0.0180 (16)	−0.0010 (11)	0.0104 (12)	0.0007 (12)
N2	0.0165 (14)	0.0137 (14)	0.0184 (16)	−0.0008 (11)	0.0093 (12)	−0.0001 (11)
N3	0.0198 (14)	0.0151 (14)	0.0198 (17)	−0.0012 (11)	0.0096 (12)	−0.0017 (12)
C1	0.0181 (16)	0.0141 (16)	0.0151 (18)	−0.0028 (13)	0.0058 (13)	−0.0019 (13)
C2	0.0207 (17)	0.0148 (16)	0.0153 (18)	−0.0004 (13)	0.0085 (14)	−0.0014 (13)
C3	0.0163 (16)	0.0154 (16)	0.0194 (19)	−0.0008 (12)	0.0088 (14)	−0.0010 (13)
C4	0.0168 (16)	0.0143 (16)	0.0160 (18)	−0.0014 (12)	0.0069 (13)	−0.0002 (13)
C5	0.0189 (17)	0.0177 (16)	0.0185 (19)	−0.0005 (13)	0.0090 (14)	−0.0028 (14)
C6	0.0178 (16)	0.0192 (17)	0.0145 (18)	−0.0005 (13)	0.0086 (14)	−0.0034 (13)
C7	0.0190 (17)	0.0208 (17)	0.0160 (19)	−0.0027 (13)	0.0072 (14)	−0.0004 (14)
C8	0.031 (2)	0.0214 (19)	0.038 (3)	−0.0080 (16)	0.0197 (19)	0.0015 (17)
C9	0.034 (2)	0.022 (2)	0.040 (3)	−0.0068 (17)	0.013 (2)	0.0059 (18)
C10	0.0263 (19)	0.0199 (18)	0.038 (2)	0.0011 (15)	0.0207 (18)	−0.0050 (17)
C11	0.0191 (16)	0.0188 (16)	0.0097 (17)	0.0032 (13)	0.0084 (13)	0.0027 (13)
C12	0.0187 (17)	0.0179 (17)	0.0174 (19)	0.0013 (13)	0.0074 (14)	0.0022 (14)
C13	0.0302 (19)	0.0203 (17)	0.0136 (18)	0.0050 (15)	0.0105 (15)	0.0018 (14)
C14	0.0262 (19)	0.028 (2)	0.026 (2)	0.0118 (16)	0.0168 (17)	0.0062 (17)
C15	0.0183 (17)	0.030 (2)	0.027 (2)	0.0033 (15)	0.0113 (16)	0.0083 (17)
C16	0.0192 (17)	0.0217 (18)	0.024 (2)	−0.0005 (14)	0.0090 (15)	0.0037 (15)
C17	0.0179 (16)	0.0142 (16)	0.0179 (19)	0.0006 (13)	0.0084 (14)	0.0016 (13)
C18	0.0223 (18)	0.0159 (16)	0.024 (2)	−0.0039 (14)	0.0097 (15)	−0.0008 (14)
C19	0.0252 (19)	0.0219 (18)	0.026 (2)	−0.0018 (15)	0.0141 (16)	0.0015 (15)
C20	0.0195 (17)	0.0170 (17)	0.027 (2)	−0.0014 (13)	0.0115 (15)	0.0067 (15)
C21	0.0246 (18)	0.0139 (16)	0.026 (2)	−0.0033 (14)	0.0094 (16)	−0.0010 (15)
C22	0.0265 (19)	0.0164 (17)	0.021 (2)	−0.0020 (14)	0.0121 (16)	−0.0028 (14)

### Geometric parameters (Å, °)

**Table:**

S1—C4	1.719 (3)	C9—H9B	0.9600
S1—C6	1.727 (3)	C9—H9C	0.9600
Cl1—C20	1.747 (4)	C10—H10A	0.9600
F1—C14	1.360 (4)	C10—H10B	0.9600
O1—C7	1.347 (4)	C10—H10C	0.9600
O1—C8	1.456 (4)	C11—C12	1.389 (5)
O2—C7	1.207 (4)	C11—C16	1.389 (5)
N1—C3	1.334 (4)	C12—C13	1.389 (5)
N1—N2	1.356 (4)	C12—H12A	0.9300
N2—C1	1.378 (4)	C13—C14	1.375 (5)
N2—C11	1.429 (4)	C13—H13A	0.9300
N3—C4	1.319 (4)	C14—C15	1.380 (6)
N3—C5	1.378 (4)	C15—C16	1.393 (5)
C1—C2	1.374 (5)	C15—H15A	0.9300
C1—C17	1.478 (5)	C16—H16A	0.9300
C2—C3	1.408 (5)	C17—C18	1.394 (5)
C2—H2A	0.9300	C17—C22	1.399 (5)
C3—C4	1.457 (5)	C18—C19	1.389 (5)
C5—C6	1.373 (5)	C18—H18A	0.9300
C5—C10	1.498 (5)	C19—C20	1.387 (5)
C6—C7	1.467 (5)	C19—H19A	0.9300
C8—C9	1.507 (6)	C20—C21	1.378 (6)
C8—H8A	0.9700	C21—C22	1.394 (5)
C8—H8B	0.9700	C21—H21A	0.9300
C9—H9A	0.9600	C22—H22A	0.9300
			
C4—S1—C6	88.81 (17)	C5—C10—H10B	109.5
C7—O1—C8	116.8 (3)	H10A—C10—H10B	109.5
C3—N1—N2	104.8 (3)	C5—C10—H10C	109.5
N1—N2—C1	111.8 (3)	H10A—C10—H10C	109.5
N1—N2—C11	118.3 (3)	H10B—C10—H10C	109.5
C1—N2—C11	129.8 (3)	C12—C11—C16	120.9 (3)
C4—N3—C5	110.6 (3)	C12—C11—N2	118.1 (3)
C2—C1—N2	106.4 (3)	C16—C11—N2	121.0 (3)
C2—C1—C17	128.9 (3)	C13—C12—C11	119.6 (3)
N2—C1—C17	124.7 (3)	C13—C12—H12A	120.2
C1—C2—C3	105.1 (3)	C11—C12—H12A	120.2
C1—C2—H2A	127.4	C14—C13—C12	118.5 (4)
C3—C2—H2A	127.4	C14—C13—H13A	120.8
N1—C3—C2	111.8 (3)	C12—C13—H13A	120.8
N1—C3—C4	119.4 (3)	F1—C14—C13	118.2 (4)
C2—C3—C4	128.7 (3)	F1—C14—C15	118.6 (4)
N3—C4—C3	123.1 (3)	C13—C14—C15	123.2 (3)
N3—C4—S1	115.5 (3)	C14—C15—C16	118.1 (3)
C3—C4—S1	121.4 (3)	C14—C15—H15A	120.9
C6—C5—N3	114.5 (3)	C16—C15—H15A	120.9
C6—C5—C10	126.7 (3)	C11—C16—C15	119.6 (4)
N3—C5—C10	118.8 (3)	C11—C16—H16A	120.2
C5—C6—C7	127.6 (3)	C15—C16—H16A	120.2
C5—C6—S1	110.6 (3)	C18—C17—C22	119.1 (3)
C7—C6—S1	121.8 (3)	C18—C17—C1	121.8 (3)
O2—C7—O1	124.5 (3)	C22—C17—C1	119.0 (3)
O2—C7—C6	124.8 (3)	C19—C18—C17	120.5 (3)
O1—C7—C6	110.7 (3)	C19—C18—H18A	119.8
O1—C8—C9	107.1 (3)	C17—C18—H18A	119.8
O1—C8—H8A	110.3	C20—C19—C18	118.9 (4)
C9—C8—H8A	110.3	C20—C19—H19A	120.6
O1—C8—H8B	110.3	C18—C19—H19A	120.6
C9—C8—H8B	110.3	C21—C20—C19	122.2 (3)
H8A—C8—H8B	108.6	C21—C20—Cl1	119.5 (3)
C8—C9—H9A	109.5	C19—C20—Cl1	118.3 (3)
C8—C9—H9B	109.5	C20—C21—C22	118.4 (3)
H9A—C9—H9B	109.5	C20—C21—H21A	120.8
C8—C9—H9C	109.5	C22—C21—H21A	120.8
H9A—C9—H9C	109.5	C21—C22—C17	120.9 (4)
H9B—C9—H9C	109.5	C21—C22—H22A	119.6
C5—C10—H10A	109.5	C17—C22—H22A	119.6
			
C3—N1—N2—C1	0.2 (4)	C5—C6—C7—O1	−176.2 (4)
C3—N1—N2—C11	177.3 (3)	S1—C6—C7—O1	2.9 (4)
N1—N2—C1—C2	−0.2 (4)	C7—O1—C8—C9	154.3 (4)
C11—N2—C1—C2	−176.9 (3)	N1—N2—C11—C12	−34.4 (5)
N1—N2—C1—C17	177.8 (3)	C1—N2—C11—C12	142.0 (4)
C11—N2—C1—C17	1.1 (6)	N1—N2—C11—C16	145.5 (3)
N2—C1—C2—C3	0.1 (4)	C1—N2—C11—C16	−38.0 (5)
C17—C1—C2—C3	−177.8 (4)	C16—C11—C12—C13	1.8 (5)
N2—N1—C3—C2	−0.1 (4)	N2—C11—C12—C13	−178.2 (3)
N2—N1—C3—C4	178.5 (3)	C11—C12—C13—C14	1.0 (5)
C1—C2—C3—N1	0.0 (4)	C12—C13—C14—F1	178.4 (3)
C1—C2—C3—C4	−178.5 (4)	C12—C13—C14—C15	−2.1 (6)
C5—N3—C4—C3	179.1 (3)	F1—C14—C15—C16	179.8 (3)
C5—N3—C4—S1	0.0 (4)	C13—C14—C15—C16	0.3 (6)
N1—C3—C4—N3	−174.4 (3)	C12—C11—C16—C15	−3.6 (6)
C2—C3—C4—N3	4.0 (6)	N2—C11—C16—C15	176.5 (3)
N1—C3—C4—S1	4.7 (5)	C14—C15—C16—C11	2.5 (6)
C2—C3—C4—S1	−176.9 (3)	C2—C1—C17—C18	123.4 (4)
C6—S1—C4—N3	0.4 (3)	N2—C1—C17—C18	−54.1 (5)
C6—S1—C4—C3	−178.8 (3)	C2—C1—C17—C22	−55.6 (5)
C4—N3—C5—C6	−0.4 (5)	N2—C1—C17—C22	126.9 (4)
C4—N3—C5—C10	178.2 (3)	C22—C17—C18—C19	−0.2 (5)
N3—C5—C6—C7	179.9 (3)	C1—C17—C18—C19	−179.2 (3)
C10—C5—C6—C7	1.4 (7)	C17—C18—C19—C20	0.2 (6)
N3—C5—C6—S1	0.7 (4)	C18—C19—C20—C21	−0.1 (6)
C10—C5—C6—S1	−177.8 (3)	C18—C19—C20—Cl1	178.8 (3)
C4—S1—C6—C5	−0.6 (3)	C19—C20—C21—C22	0.0 (6)
C4—S1—C6—C7	−179.8 (3)	Cl1—C20—C21—C22	−178.9 (3)
C8—O1—C7—O2	−2.1 (6)	C20—C21—C22—C17	0.0 (6)
C8—O1—C7—C6	178.0 (3)	C18—C17—C22—C21	0.1 (5)
C5—C6—C7—O2	3.9 (6)	C1—C17—C22—C21	179.1 (3)
S1—C6—C7—O2	−177.0 (3)		

### Hydrogen-bond geometry (Å, °)

**Table:**

*D*—H···*A*	*D*—H	H···*A*	*D*···*A*	*D*—H···*A*
C15—H15A···O2^i^	0.93	2.48	3.251 (5)	141

Symmetry codes: (i) *x*−1, −*y*+1/2, *z*+1/2.
